# Supplementary material for: Optimal planning and partitioning of multiple distribution Micro-Grids based on reliability evaluation
Source: PLoS One. 2024 Jul 31;19(7):e0305745. doi: 10.1371/journal.pone.0305745 (PMC11290660; doi:10.1371/journal.pone.0305745)
Supplement: Attachment — Submitted filename: PONE-D-15840-Comments.docx [file pone.0305745.s002.docx]

**PONE-D-24-15840-peer-review-v1**

**Optimal planning and partitioning of multiple distribution micro-grids based on reliability evaluation**

This paper proposes a stochastic optimization for microgrid planning. I have the following comments for your consideration.

1. The abstract should be double-checked for typos, as the presence of "candida" seems glaringly incorrect.
2. The approach to handling uncertainty through the "probability tree" method appears to lack sufficient innovation.
3. It would be beneficial to further justify on the choice of genetic algorithms for problem-solving. Comparative analysis with established tools like Gurobi and CPLEX could strengthen the justification for the proposed calculation method.
4. The introduction would benefit from more recent references to enhance the review's currency.
5. Improvement in the font size and resolution of figures 2 and 6 is recommended for better readability.
6. Have you considered the operational stage to validate the investment decisions?
7. Could you demonstrate on how and at what stage the effect of operational risks is justified?
