# Supplementary material for: Optimal planning and partitioning of multiple distribution Micro-Grids based on reliability evaluation
Source: PLoS One. 2024 Jul 31;19(7):e0305745. doi: 10.1371/journal.pone.0305745 (PMC11290660; doi:10.1371/journal.pone.0305745)
Supplement: Attachment — Submitted filename: Response to reviewers_V1.doc [file pone.0305745.s003.doc]

Dear Editor,

Thank you so much for sending the reviewers comments related to manuscript entitled “Optimal Planning and Partitioning of Multiple Distribution Micro-Grids Based on Reliability Evaluation”. We applied the reviewers’ comments to improve the quality of the paper. Responses to the reviewers’ comments are presented in the same order as the comments have been addressed.

Reviewer #1:

Thank you so much for your time and valuable comments. Based on your comments, the paper has been revised and edited. The revisions made according to your comments are as followed:

1) The abstract needs to be restructured for clarity and to emphasize the novelty of the research. The current version of the abstract is too lengthy and may benefit from a more concise presentation to effectively communicate the key findings and contributions of the study.

**Answer:**

According to this comment, the abstract has been shortened and edited as follows:

**Edited text (section abstract):**

Power system researcher have turned to Micro-Grids (MG) for higher reliability, greater flexibility, lower operating costs and losses, and lower CO_2_ emissions at the distribution system. This paper presents a single-level stochastic optimization framework for planning and partitioning of a distribution system including Multiple Micro-Grids (MMGs). The main objective is to minimize the total cost of the system including investment, operation, total losses and reliability costs of the distribution network. The proposed model takes into account the viewpoints of MG owners and distribution system operators, simultaneously. The voltage stability index is introduced to identify the optimal site of MG investment. To deal with uncertainties caused by renewable generations, the Firefly Algorithm (FA) and probability-tree method is used to create various operation scenarios of Photo-Voltaic (PVs) and Wind Turbines (WTs). This model is solved through the genetic algorithm in MATLAB, and to evaluate its effectiveness, numerical studies have been carried out on the experimental IEEE distribution network with four specified locations for investing MGs and seven Tie Switches (TS) for network partitioning. Simulation results reveal that optimal locations for MG investment are determined in such a way all MGs connect to the buses near the beginning of the feeder and as a result, load point reliability is improved, total active power losses are reduced, and the energy program becomes more optimized.

2) The authors have reviewed previous studies that investigate multi-objective modelling. Could they clarify why multiple objectives were not considered in the present study?

**Answer:**

As stated in this article, several objectives have been used to optimize the problem. The text below describes the article in this context and the additional corrections of this comment have been added to the relevant section.

**Edited text (section 2.1):**

The main objective is to minimize the total cost of the system including investment, operation, total losses and reliability costs of the distribution network. Mathematical modelling of MMGs Planning and partitioning includes the objective function and technical-economic constraints. Here, the objective function is defined as (1).

| $Min\mathop{}\limits(of_{Planning}^{MMGs}=TC)\to TC=\left\{\sum_{MGi=1}^{Nmg} C_{Inv}^{MGi}+\sum_{SWi=1}^{Nsw} C_{Inv}^{SWi}\right\}+C_{O}^{DS,Ex}$ | (1) |
| --- | --- |

In the objective function, there are two terms. The first one is the sum of investment costs for all MGs and TSs in the distribution system, and the second one is the expected operation costs of the distribution system considering various operating scenarios. …

3) The authors utilized stochastic optimization to handle uncertainty. It would be beneficial for them to compare stochastic optimization with existing uncertainty handling methods to highlight the advantages of their approach.

**Answer:**

In the article, these structures mentioned in the works of other authors are mentioned in detail. But there has been no work that exactly corresponds to the data of this article and has used method 1 so that it can be compared with the present article. However, in this article, the results are compared.

4) Adding a system structure in section 2 is recommended.

**Answer:**

According to this comment, system structure and Overall procedure of solving the proposed problem has been added as follows:

**Added text (section 2):**

Fig. 1 shows the structure of the system investigated in this paper. According to this figure, the components of this system are shown to be connected to the MGs.

**Figure 1:** System structure

Also, Fig. 2 shows the overall procedure of solving the proposed problem of this article. In the following, each of the steps will be explained in detail.

**Figure 2:** Overall procedure of solving the proposed problem

5) There is a notable need to enhance the quality of figures and tables presented in the paper, as they currently appear suboptimal.

**Answer:**

According to this comment, font size and resolution of figures 2, 6 and 9 has been revised. The quality of the tables has also been improved.

6) The size of the equations is too large and should be adjusted for better readability.

**Answer:**

According to this comment, the size of equations has been reduced and has reached the standard value.

7) The study lacks enough recent references, which is crucial for reflecting the latest advancements in the research topic. More references from the last two years are recommended.

**Answer:**

According to this comment, new references have been added to the introduction of the paper and corrections have been made as follows.

**Added and edited text (section introduction):**

Reference [3] investigates a method for clustering the traditional passive distribution system in a cluster of MMGs network. Among the benefits of traditional distribution system clustering in MMG for customers and distribution system operators include local control strategy to minimize interaction between different MGs, local reactive power compensation to prevent fault propagation, minimize power losses and ultimately improve network reliability. Reference [4] also provides a suitable model for strengthening the security of MMG-based active distribution networks by optimally using consumption management plans and energy storage resources. In the proposed model, a hierarchical two-stage approach has been established in the form of an incident and its impact on the distribution network and preventive and corrective measures to increase system readiness and reduce damages caused by severe accidents.

A parallel computing framework based on spectral partitioning aimed at increasing the computational efficiency of large-scale microscopic traffic simulation is introduced in [7]. This framework uses the SP method to segment road networks, considering vehicle information and road information as building blocks to determine node weights.

The topology of flexible interconnected distribution network is analyzed according to [12] and the concept of partition and block is proposed. The flexible interconnected distribution network of this reference is divided into three areas: DC, AC and SOP. Based on the partition, Dijkstra's algorithm is used to search for the shortest circuit to form the block, and the block information of each area is specified.

To control the risk of renewable resources with high volatility, a risk-based model for optimal planning and segmentation of MMGs considering the uncertainty of renewable resources is presented in [26]. In addition, the term techno-economic risk has been introduced to analyze the total risk in MMG. Also, the energy sources of distributed generation and storage in MGs and the state of connection switches to connect MGs to the network are determined simultaneously.

[3] Sireesha R, Srinivasa Rao C, Vijay Kumar M. Graph theory based transformation of existing Distribution network into clusters of multiple micro-grids for reliability enhancement. Materials Today: Proceedings. 2023;80(3): 2921-28.

[4] Ahrari M, Shirini K, Gharehveran SS, Ahsaee MG, Haidari S, Anvari P. A security-constrained robust optimization for energy management of active distribution networks with presence of energy storage and demand flexibility. Journal of Energy Storage. 2024;84(B). https://doi.org/10.1016/j.est.2024.111024.

[7] Liu Z, Xie S, Zhang H, Zhou D, Yang Y. A parallel computing framework for large-scale microscopic traffic simulation based on spectral partitioning. Transportation Research Part E: Logistics and Transportation Review. 2024;181. <https://doi.org/10.1016/j.tre.2023.103368>.

[12] Chen X, Zhao Y, Xiong W, Yuan X, Zou X. Partition and Block Algorithm for Active Distribution Network Reliability Evaluation with a Soft Open Point. 2023 5th Asia Energy and Electrical Engineering Symposium (AEEES), Chengdu, China. 2023; 777-783. 10.1109/AEEES56888.2023.10114172.

[26] Mortazavi N, Alizadeh B, Sahab A, Barzegar SMS. Risk-Based planning and partitioning of multiple micro-grids in distribution systems. International Journal of Electrical Power & Energy Systems. 2022;137. <https://doi.org/10.1016/j.ijepes.2021.107659>.

Reviewer #2:

Thank you so much for your time and valuable comments. Based on your comments, the paper has been revised and edited. The revisions made according to your comments are as followed:

1) The abstract should be double-checked for typos, as the presence of "candida" seems glaringly incorrect.

**Answer:**

According to this comment, the abstract has been edited as follows:

**Edited text (section abstract):**

Power system researcher have turned to Micro-Grids (MG) for higher reliability, greater flexibility, lower operating costs and losses, and lower CO_2_ emissions at the distribution system. This paper presents a single-level stochastic optimization framework for planning and partitioning of a distribution system including Multiple Micro-Grids (MMGs). The main objective is to minimize the total cost of the system including investment, operation, total losses and reliability costs of the distribution network. The proposed model takes into account the viewpoints of MG owners and distribution system operators, simultaneously. The voltage stability index is introduced to identify the optimal site of MG investment. To deal with uncertainties caused by renewable generations, the Firefly Algorithm (FA) and probability-tree method is used to create various operation scenarios of Photo-Voltaic (PVs) and Wind Turbines (WTs). This model is solved through the genetic algorithm in MATLAB, and to evaluate its effectiveness, numerical studies have been carried out on the experimental IEEE distribution network with four specified locations for investing MGs and seven Tie Switches (TS) for network partitioning. Simulation results reveal that optimal locations for MG investment are determined in such a way all MGs connect to the buses near the beginning of the feeder and as a result, load point reliability is improved, total active power losses are reduced, and the energy program becomes more optimized.

2) The approach to handling uncertainty through the "probability tree" method appears to lack sufficient innovation.

**Answer:**

In addition to probability tree, the proposed method of the article solves the problem based on Firefly Algorithm (FA), which is the innovation of the article and its results also show the accuracy and improvement in solving the problem. This innovation has been added to the abstract and introduction section of the article.

**Added text (section abstract):**

To deal with uncertainties caused by renewable generations, the Firefly Algorithm (FA) and probability-tree method is used to create various operation scenarios of Photo-Voltaic (PVs) and Wind Turbines (WTs).

**Added text (section introduction):**

By reviewing the references and stating the goals of each one, the summary of the work done and its comparison with the innovations of this paper is shown in Table (1).

Table (1): Compare the proposed method and previous methods

| **No.** | **Aspect** | **[3]** | **[4]** | **[18]** | **[19]** | **[22]** | **[23]** | **[24]** | **[25]** | **[26]** | **[27]** | **[28]** | **[29]** | **[30]** | **This paper** |
| --- | --- | --- | --- | --- | --- | --- | --- | --- | --- | --- | --- | --- | --- | --- | --- |
| **1** | Partitioning | ✓ | ✓ |  |  |  | |  |  | ✓ |  |  |  |  | ✓ |
| **2** | Uncertainty |  | ✓ | ✓ | ✓ | ✓ | | ✓ | ✓ | ✓ |  |  |  |  | ✓ |
| **3** | Equipment Aging |  | ✓ |  |  |  | |  |  |  |  | ✓ | ✓ | ✓ |  |
| **4** | DM Algorithm |  |  |  |  | ✓ | |  |  |  |  | ✓ | ✓ | ✓ |  |
| **5** | MG Clustering | ✓ | ✓ |  |  |  | |  |  |  | ✓ |  |  |  |  |
| **6** | Opt. Algorithm |  | ✓ | ✓ |  | ✓ | |  |  |  | ✓ |  |  |  | ✓ |
| **7** | MMG | ✓ | ✓ |  | ✓ | ✓ | |  |  | ✓ |  |  |  |  | ✓ |
| **8** | Prediction |  |  | ✓ | ✓ |  | | ✓ | ✓ |  |  |  |  |  | ✓ |
| **9** | Reliability | ✓ | ✓ |  |  |  | |  |  |  |  |  |  |  | ✓ |
| **10** | FA |  |  |  |  |  | |  |  |  |  |  |  |  | ✓ |
| **11** | expected costs of operation |  |  |  |  |  | |  |  |  |  |  |  |  | ✓ |
| **12** | VSI |  |  |  |  |  | |  |  |  |  |  |  |  | ✓ |
| **13** | probability-tree method |  |  |  |  |  | |  |  |  |  |  |  |  | ✓ |
| DM: Decision-Making MG: Micro-Grid Opt.: Optimization  MMG: Multi Micro-Grid FA Firefly Algorithm VSI: Voltage Stability Index | | | | | | | | | | | | | | | |

3) It would be beneficial to further justify on the choice of genetic algorithms for problem-solving. Comparative analysis with established tools like Gurobi and CPLEX could strengthen the justification for the proposed calculation method.

**Answer:**

Thanks for this very appropriate comment. The problem solving method is based on FA Algorithm. Based on this, the main focus of this article is on this solution method and the results obtained from it, and as can be seen from the results, the accuracy of the outputs is evident and the improvement of the problem is clear. Now this issue and comparison with other algorithms can be introduced as a suggestion to continue this work.

4) The introduction would benefit from more recent references to enhance the review's currency.

**Answer:**

According to this comment, new references have been added to the introduction of the paper and corrections have been made as follows.

**Added and edited text (section introduction):**

Reference [3] investigates a method for clustering the traditional passive distribution system in a cluster of MMGs network. Among the benefits of traditional distribution system clustering in MMG for customers and distribution system operators include local control strategy to minimize interaction between different MGs, local reactive power compensation to prevent fault propagation, minimize power losses and ultimately improve network reliability. Reference [4] also provides a suitable model for strengthening the security of MMG-based active distribution networks by optimally using consumption management plans and energy storage resources. In the proposed model, a hierarchical two-stage approach has been established in the form of an incident and its impact on the distribution network and preventive and corrective measures to increase system readiness and reduce damages caused by severe accidents.

A parallel computing framework based on spectral partitioning aimed at increasing the computational efficiency of large-scale microscopic traffic simulation is introduced in [7]. This framework uses the SP method to segment road networks, considering vehicle information and road information as building blocks to determine node weights.

The topology of flexible interconnected distribution network is analyzed according to [12] and the concept of partition and block is proposed. The flexible interconnected distribution network of this reference is divided into three areas: DC, AC and SOP. Based on the partition, Dijkstra's algorithm is used to search for the shortest circuit to form the block, and the block information of each area is specified.

To control the risk of renewable resources with high volatility, a risk-based model for optimal planning and segmentation of MMGs considering the uncertainty of renewable resources is presented in [26]. In addition, the term techno-economic risk has been introduced to analyze the total risk in MMG. Also, the energy sources of distributed generation and storage in MGs and the state of connection switches to connect MGs to the network are determined simultaneously.

[3] Sireesha R, Srinivasa Rao C, Vijay Kumar M. Graph theory based transformation of existing Distribution network into clusters of multiple micro-grids for reliability enhancement. Materials Today: Proceedings. 2023;80(3): 2921-28.

[4] Ahrari M, Shirini K, Gharehveran SS, Ahsaee MG, Haidari S, Anvari P. A security-constrained robust optimization for energy management of active distribution networks with presence of energy storage and demand flexibility. Journal of Energy Storage. 2024;84(B). https://doi.org/10.1016/j.est.2024.111024.

[7] Liu Z, Xie S, Zhang H, Zhou D, Yang Y. A parallel computing framework for large-scale microscopic traffic simulation based on spectral partitioning. Transportation Research Part E: Logistics and Transportation Review. 2024;181. <https://doi.org/10.1016/j.tre.2023.103368>.

[12] Chen X, Zhao Y, Xiong W, Yuan X, Zou X. Partition and Block Algorithm for Active Distribution Network Reliability Evaluation with a Soft Open Point. 2023 5th Asia Energy and Electrical Engineering Symposium (AEEES), Chengdu, China. 2023; 777-783. 10.1109/AEEES56888.2023.10114172.

[26] Mortazavi N, Alizadeh B, Sahab A, Barzegar SMS. Risk-Based planning and partitioning of multiple micro-grids in distribution systems. International Journal of Electrical Power & Energy Systems. 2022;137. <https://doi.org/10.1016/j.ijepes.2021.107659>.

5) Improvement in the font size and resolution of figures 2 and 6 is recommended for better readability.

**Answer:**

According to this comment, font size and resolution of figures 2, 6 and 9 has been revised.

6) Have you considered the operational stage to validate the investment decisions?

**Answer:**

The main objective of this paper is to minimize the total cost of the system including investment, operation, total losses and reliability costs of the distribution network. For considered the operational stage, one year is divided into four seasons and also each period for operation planning study is equal to three months. The expected cost of the distribution network in each operation planning period can be computed according to relation:

7) Could you demonstrate on how and at what stage the effect of operational risks is justified?

**Answer:**

According to the results of this article, total MMGs risk is optimally evaluated as equal to 2415 dollars. Total planning and operation cost for year1, year2, year3 and year4 are evaluated 210555$, 255642 $, 287323 $ and 302101 $, respectively.
